# Synthesis of Radiopharmaceuticals *via* “In-Loop” ^11^C-Carbonylation as Exemplified by the Radiolabeling of Inhibitors of Bruton's Tyrosine Kinase

**DOI:** 10.3389/fnume.2021.820235

**Published:** 2022-01-20

**Authors:** David J. Donnelly, Sean Preshlock, Tanpreet Kaur, Tritin Tran, Thomas C. Wilson, Karim Mhanna, Bradford D. Henderson, Daniel Batalla, Peter J. H. Scott, Xia Shao

**Affiliations:** ^1^Discovery Chemistry Platforms, PET Radiochemical Synthesis, Bristol Myers Squibb Research and Development, Princeton, NJ, United States; ^2^Department of Radiology, University of Michigan, Ann Arbor, MI, United States

**Keywords:** BTK inhibitors, carbon monoxide, carbon-11, tolebrutinib, evobrutinib, radiochemistry, positron emission tomography, ibrutinib

## Abstract

Positron emission tomography (PET) is an important non-invasive tool to help guide the drug discovery and development process. Positron-emitting–radiolabeled drug candidates represent an important tool for drug hunters to gain insight into a drug's biodistribution and target engagement of exploratory biologic targets of interest. Recently, there have been several drug candidates that incorporate an acryloyl functional group due to their ability to form a covalent bond within the biological target of interest through Michael addition. Methods to incorporate a carbon-11 radionuclide into acrylamide derivatives remain challenging given the reactive nature of this moiety. Herein, we report the improved radiosynthesis of carbon-11–containing acrylamide drug candidates, [^11^C]ibrutinib, [^11^C]tolebrutinib, and [^11^C]evobrutinib, using [^11^C]CO and a novel “in-loop” ^11^*C*-carbonylation reaction. [^11^C]Ibrutinib, [^11^C]tolebrutinib, and [^11^C]evobrutinib were reliably synthesized, generating 2.2-7.1 GBq of these radiopharmaceuticals in radiochemical yields ranging from 3.3 to 12.8% (non-decay corrected; relative to starting [^11^C]CO_2_) and molar activities of 281-500 GBq/μmol (7.5-13.5 Ci/μmol), respectively. This study highlights an improved method for incorporating carbon-11 into acrylamide drug candidates using [^11^C]CO within an HPLC loop suitable for clinical translation using simple modifications of standard automated synthesis modules used for cGMP manufacture of PET radioligands.

## Introduction

B-cells are critical to regulating immune responses in both physiological and pathological conditions. Indeed, several B-cell–mediated diseases are thought to occur due to the dysregulation of B-cell function. This includes B-cell malignancies (e.g., lymphomas, leukemias) (1), autoimmune disorders {rheumatoid arthritis (RA) (2–4), multple sclerosis (MS) (5–7), inflammatory diseases [e.g., obesity, diabetes (8, 9)]}, and transplant complications [graft *vs*. host disease (10, 11)]. Reflecting this, B-cells are an extremely important therapeutic target. One approach has involved the development of inhibitors of Bruton's tyrosine kinase (BTK) (12). BTK is a cytoplasmic tyrosine kinase expressed by B-cells as well as myeloid cells such as microglia. The kinase plays a crucial role in B-cell development, where it is involved in several signal transduction pathways (13). From a myeloid tissue perspective, BTK plays a role in mast cell activation *via* the high-affinity IgE receptor (14). Given these roles, BTK inhibitors are being evaluated for the treatment of B-cell malignancies (15), as well as for inflammatory and autoimmune disorders (16), including RA (17) and MS (18).

**Figure 1:**
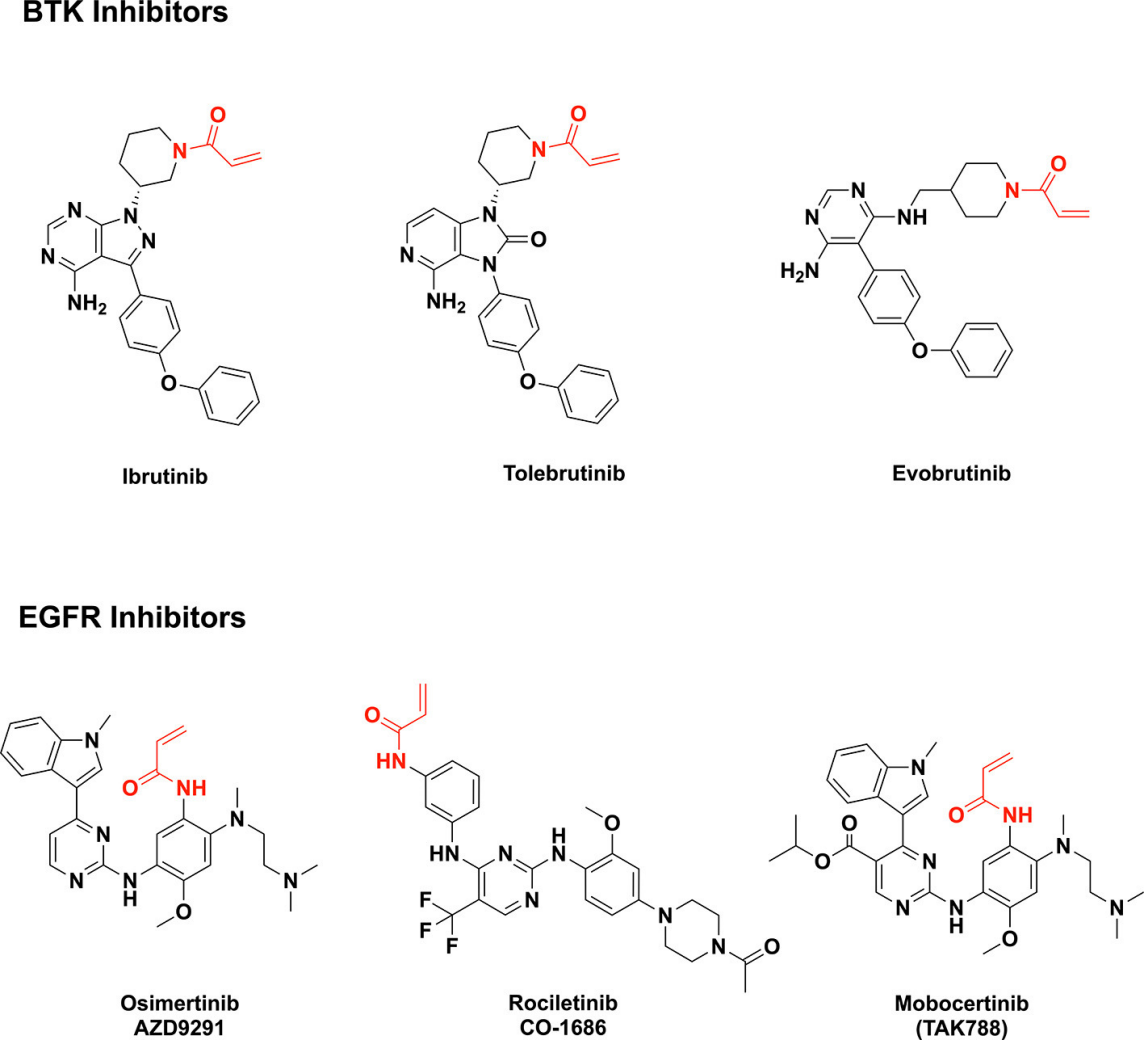
Examples of acrylamide-containing drug candidates (the acrylamide moiety is highlighted in red).

Molecular imaging is playing an increasingly important role in drug discovery and development (DDD) (19). In particular, positron emission tomography (PET) imaging is being used by drug discovery teams to ensure that effective drugs engage the right target, have limited side effects and that relevant patient populations are recruited into clinical trials, to have maximum impact on human health and lifespan. One application of PET imaging in DDD uses radiolabeled drug candidates to obtain key information about the molecule. Important information on these radiolabeled drug candidates includes (i) demonstration that the drug reaches the site of action, (ii) that it engages its intended target, (iii) identification of off-target binding, including accumulation at other sites that could lead to side effects or toxicology concerns, and (iv) key pharmacokinetic information about the drug candidates' distribution and excretion. For these reasons, in the context of B-cell mediated diseases, we and others have a particular interest in the synthesis of radiolabeled BTK inhibitors (Figure 1).

Irreversible BTK inhibitors like ibrutinib, tolebrutinib, and evobrutinib all share a common acrylamide moiety (16). Acrylamide is a pronounced Michael acceptor (20), allowing the BTK inhibitors to form covalent bonds with a cysteine residue in the BTK active site and leading to sustained inhibition of kinase activity. This is exemplified by ibrutinib, the first effective and selective BTK inhibitor approved by the U.S. Food and Drug Administration (FDA) in 2013 (for management of B-cell tumors) (16). Additionally, acrylamides are present in third-generation EGFR inhibitors such as mobovcertinib, osimertinib, and rociletinib (Figure 1) (21–23).

**Scheme 1:**
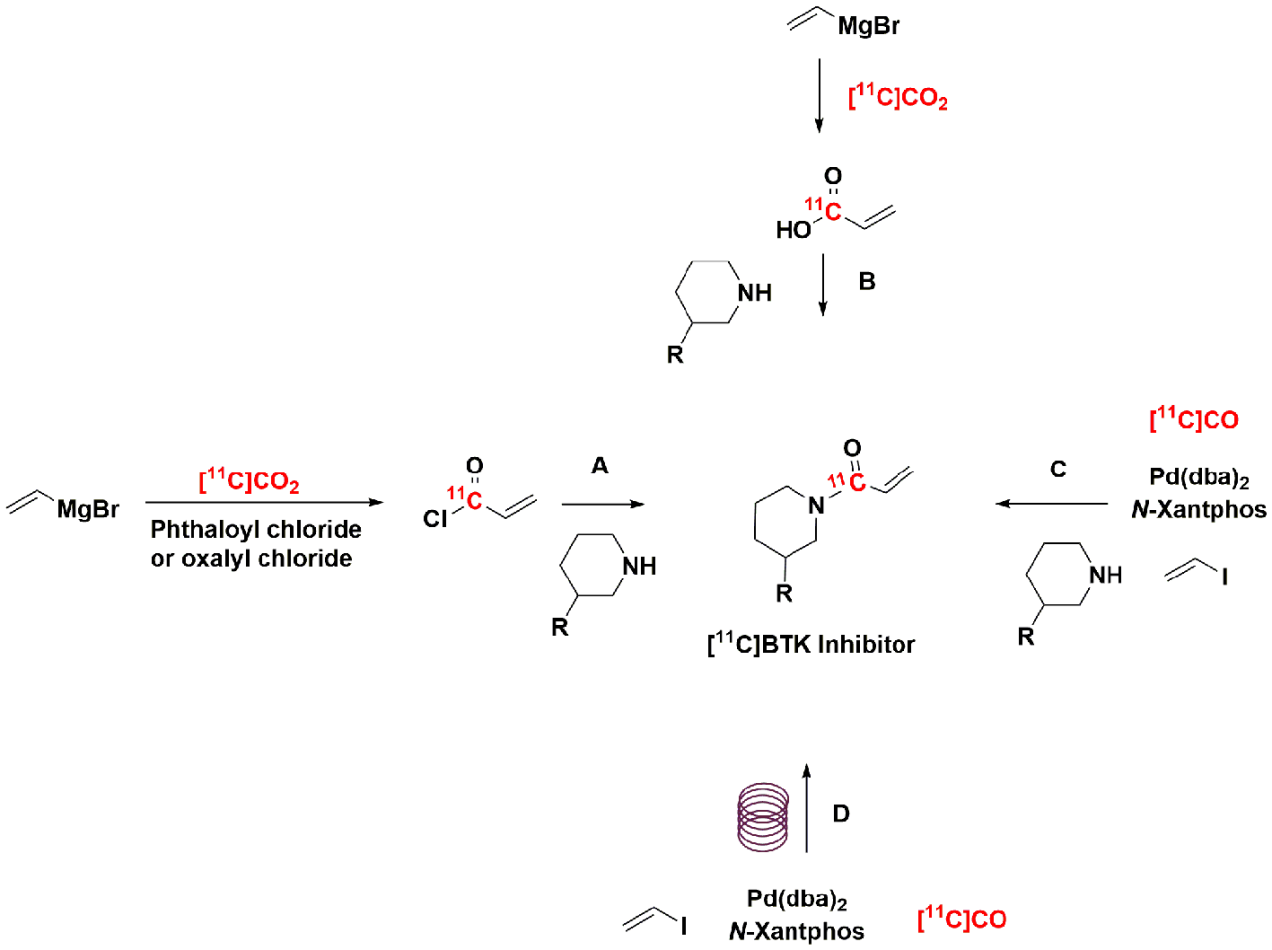
Example strategies for radiolabeling of BTK inhibitors. **(A–C)** Have been previously reported (28, 29), **(D)** this work was completed within an HPLC loop.

When considering how to prepare PET isotopologs of ibrutinib, tolebrutinib, and evobrutinib, it is necessary to utilize carbon-11 (t_1/2_ = 20 min) given the impractically short half-lives of oxygen-15 and nitrogen-13 (t_1/2_ = 2 min and 10 min, respectively). The structures of the BTK inhibitors in question offer a limited opportunity for incorporating carbon-11 into these scaffolds and, as such, we reasoned that labeling the carbonyl group of the acrylamide moiety was most likely to be successful because of synthetic accessibility of the requisite precursors and precedent for synthesizing [^11^C]acrylic acid derivatives (24–28). When our teams set out in a campaign to prepare [^11^C]ibrutinib in 2016, our best option was to use [^11^C]CO_2_ fixation to install the [^11^C]C=O group in the acrylamide moiety (Scheme 1) (29). This allowed us to prepare [^11^C]ibrutinib in sufficient radiochemical yield for initial preclinical evaluation. However, the method is problematic for several reasons. First, it is necessary to perform syntheses using carrier CO_2_ to obtain good radiochemical yields (RCY) (up to 15%) and purity (99%), but this resulted in low molar activity product (0.2 GBq /μmol, 55 mCi/ μmol). Conducting no-carrier-added syntheses *via* [^11^C]CO_2_ fixation resulted in higher molar activity product (22-77 GBq/ μmol, 600-2,100 mCi/μmol), but inconsistent and low yields (<1% non-decay corrected) as well as lower chemical and radiochemical purity (~90%) due to formation of unknown byproducts.

An attractive alternative approach to mitigate these challenges is ^11^C-carbonylation using [^11^C]CO. [^11^C]CO has been used in medical applications since the 1940s and as a building block in radiosynthesis since it was used to produce [^11^C]phosgene in 1978 (30, 31). Given that the carbonyl group is one of the most widespread functional groups present in drugs and bioactive molecules, [^11^C]CO offers enormous potential for preparing ^11^C-labeled radiopharmaceuticals. Many groups have developed [^11^C]CO into a versatile ^11^C-labeling synthon and several review articles have summarized their efforts using it to synthesize ^11^C-labeled amides, esters, carboxylic acids, ketones, ureas and, albeit less commonly, acid chlorides, carbamate esters, and aldehydes (32–37). However, ^11^C-carbonylation remains operationally complex to conduct for several reasons. Until recently, [^11^C]CO has not been readily available from most cyclotrons, it is obtained in low activity and has poor solubility, and there has not been routine equipment available for working with the reagent. The majority of work has been conducted by a few research groups using specialized, often homemade equipment, which also confounds cGMP compliance. For instance, recent examples have relied on high-pressure apparatus that is not readily transferable to the vast majority of radiochemistry production laboratories (38). Recent review articles have concluded that the lack of commercially available synthesis modules for conducting ^11^C-carbonylation reactions according to cGMP is the main reason that limited ^11^C-carbonylation protocols have been used in routine PET-tracer production and that only a few PET tracers (~4 to date)(36) have been translated to clinical use. For all of these reasons, the contributions of [^11^C]CO to radiochemistry are less established than other common synthons (e.g., [^11^C]MeI, [^11^C]MeOTf, [^11^C]CO_2_), and most publications on ^11^C-carbonylaton reactions have been chemistry oriented, focusing upon method development and mapping out the reaction scope of new methodologies.

We hypothesized that these challenges could be alleviated by leveraging the more ready availability of [^11^C]CO from modern cyclotrons (e.g., utilizing the Process Cabinet of a GE PETtrace) and developing methods that are compatible with the automated synthesis modules used for cGMP manufacture of PET radiotracers today. Thus, we and others are motivated to develop more user-friendly approaches for ^11^C-carbonylation (28, 39–41). Notably, concurrent with our efforts, Lindberg et al. demonstrated that [^11^C]ibrutinib can be prepared *via* ^11^C-carbonylation in a reactor method (40), and reported preliminary pre-clinical imaging results comparable to our earlier findings (29).

**Scheme 2:**
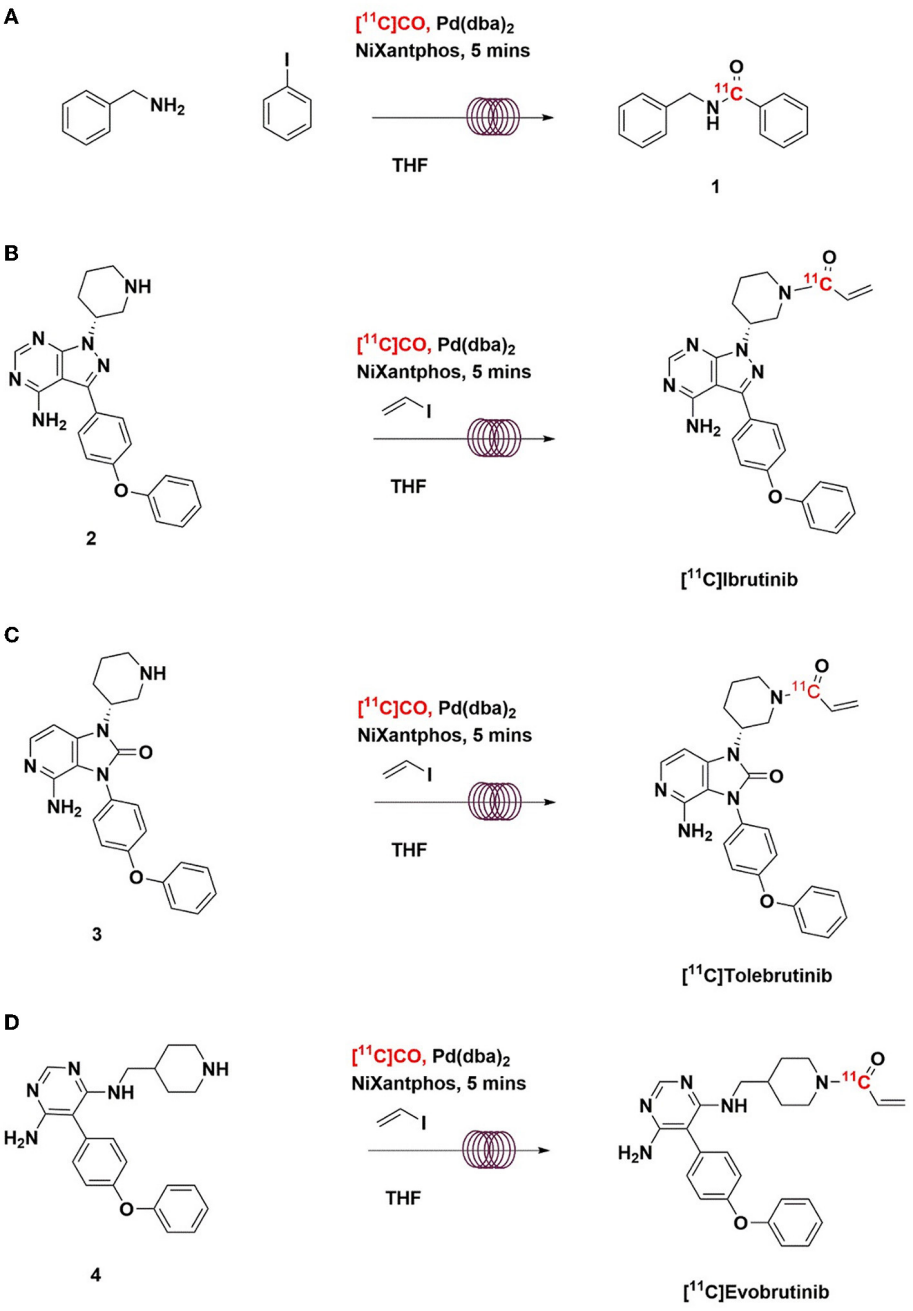
Radiosynthesis of [^11^C]*N*-benzyl benzamide **(A)**, [^11^C]Ibrutinib (B), [^11^C]Tolebrutinib **(C)**, and [^11^C]Evobrutinib **(D)** using [^11^C]CO *via* “in-loop” ^11^C-carbonylation.

In this study, we evaluated two catalysts for conversion of [^11^C]CO_2_ to [^11^C]CO (charcoal and molybdenum), and report a highly efficient method for “in-loop” ^11^C-carbonylation within a modified commercially available remote-controlled synthesis unit. The latter builds on the pioneering “in-loop” carbonylation chemistry reported by Ferrat et al. while this study was in progress (39). These approaches to ^11^C-carbonylaton have been adapted for two automated radiochemistry synthesis modules. Initial work compared them in the synthesis of a model substrate, [^11^C]N-benzyl benzamide (Scheme 2A). The model reaction established that molybdenum is the preferred catalyst for conversion of [^11^C]CO_2_ to [^11^C]CO as it provides products in high molar activity and using a simple “in-loop” ^11^C-carbonylation method. With that method in hand, we describe its use for the cGMP automated production of the radiopharmaceuticals [^11^C]ibrutinib, [^11^C]tolebrutinib, and [^11^C]evobrutinib (Schemes 2B–D) accordingly.

## Materials and Methods

### Materials

Reagents and solvents were purchased from Aldrich Chemical or Fisher Scientific and were used without further purification unless noted. Chromatography column for HPLC analysis and purification were purchased from Phenomenex or Waters. High-performance liquid chromatography (HPLC) was performed using a Shimadzu LC-2010A HT system equipped with a Bioscan B- FC-1000 radiation detector (University of Michigan) or an Agilent 1100 HPLC equipped with a lab logic posiram radio-HPLC detector (Bristol Myers Squibb). Iodoethylene was acquired from Oakwood Chemicals (SC, United States). Tetrahydrofuran (THF) anhydrous, ≥99.9%, inhibitor-free, was obtained from Sigma Aldrich (p/n 401757). Sodium chloride, 0.9% USP, and sterile water for Injection; USP was purchased from Hospira; Dehydrated Alcohol for Injection, USP was obtained from Akorn Inc. Sterile filters were acquired from Millipore; 10 cc sterile vials were obtained from HollisterStier; C18 Sep-Paks were purchased from Waters Corporation and were flushed with 10 ml of ethanol followed by 10 ml of sterile water before use. 3-(4-Phenoxyphenyl)-1-(piperidin-3-yl)-2,3-dihydro-1H-pyrazolo[3,4-d]pyrimidin-4-amine (2), (R)-4-amino-3-(4-phenoxyphenyl)-1-(piperidin-3-yl)-1,3-dihydro-2H-imidazo[4,5-c]pyridin-2-one (3), and 5-(4-phenoxyphenyl)-N4-(piperidin-4-ylmethyl)pyrimidine-4,6-diamine (4), as well as the associated non-radioactive reference standards were synthesized using standard methods described within the literature (28, 42, 43).

### Modifications

**Figure 2:**
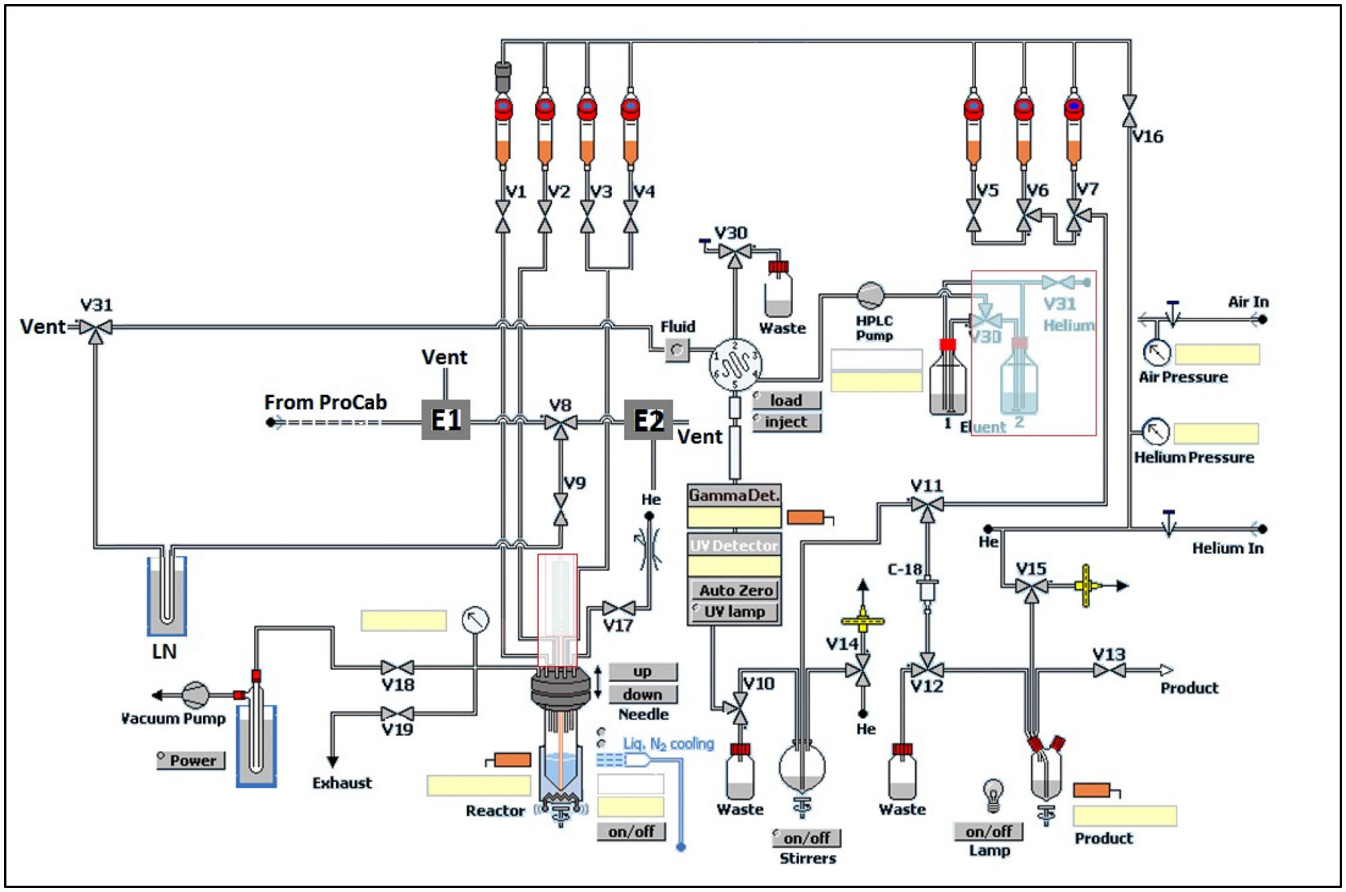
Modification of GE TracerLab FX_M_ for ^11^C-carbonylation.

**Figure 3:**
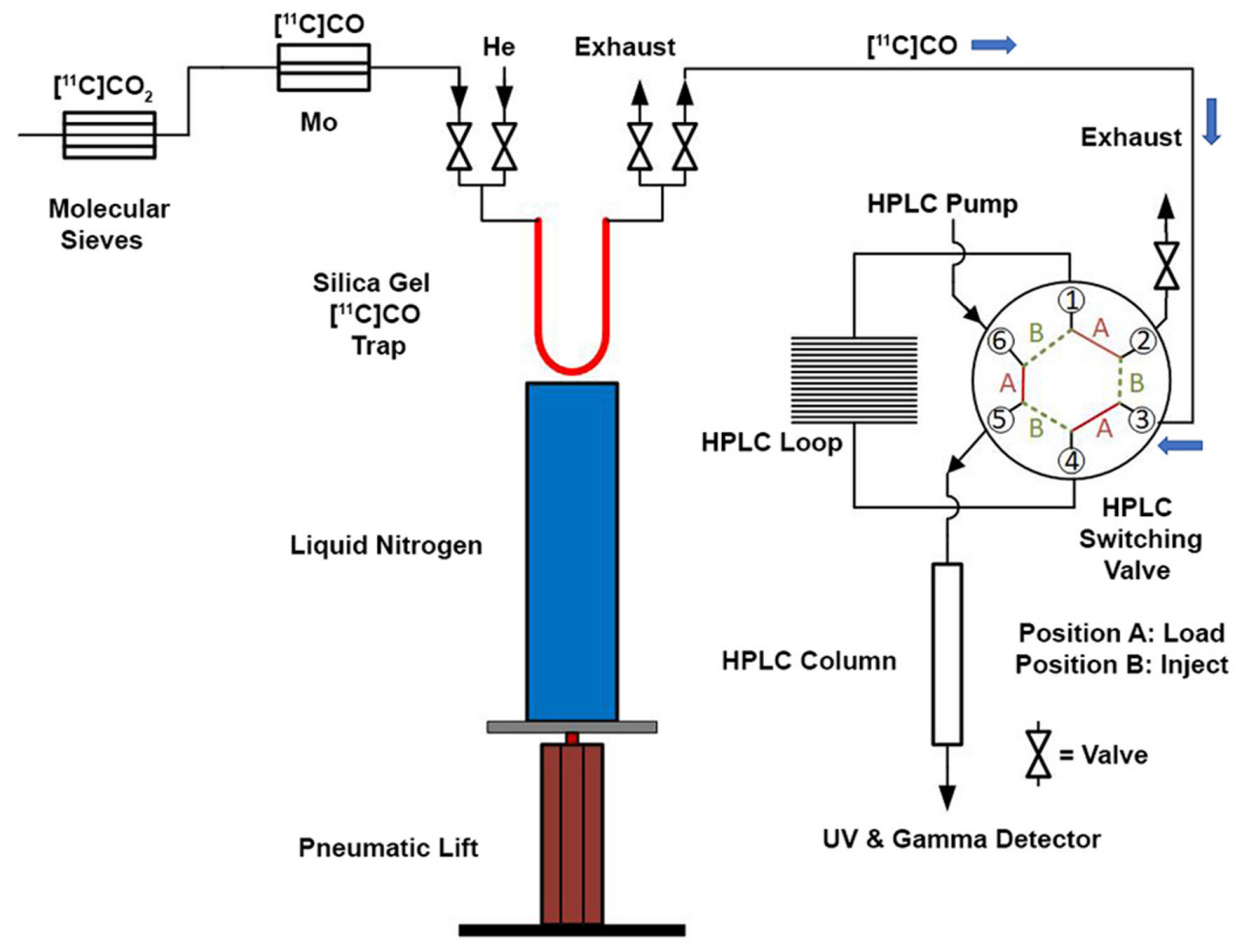
Schematic for “in-loop” ^11^C-Carbonylation. [^11^C]CO_2_ was produced and delivered to a molecular sieves column. After target delivery, [^11^C]CO_2_ was converted [^11^C]CO at 850°C and delivered to a silica gel trap at −196 °C at a flow rate of 50 mL/min. After trapping, the [^11^C]CO was transferred from the silica gel trap and this trap was warmed to ambient temperature using a pneumatic lift (or manually—see Supplementary Material). [^11^C]CO was then transferred using helium gas at a flow rate of 5 mL/min into an HPLC switching valve in position A. The ^11^C-carbonylation occurred within a sealed HPLC loop and the final ^11^C-labeled compound was further purified by activating the HPLC switching valve to position B.

Modifications of GE TracerLab FX_M_ and FX_C−Pro_ synthesis modules were made to accommodate an in-loop ^11^C-carbonylation as shown in Figure 2. To direct the gas flow, two additional electronic valves (E1 and E2) were installed and connected to each end of V8. V9 was disconnected from the needle and connected to the silica trap for [^11^C]CO. V30 and V31 were removed from the HPLC pump and connected to each end of the HPLC loop. [^11^C]CO was delivered from a General Electric Medical System (GEMS) Process Cabinet through E1, V8 (left), V9, and trapped by a silica trap (100 mg silica gel in a 1/8 inch tube) immersed in liquid nitrogen. Following completed entrapment, both valves E1 and V8 were switched, and then the silica trap was moved out of the liquid nitrogen. The [^11^C]CO was released and pushed by helium at 5 ml/min through E2, V8 (right), V9, V31, and into an HPLC loop (stainless steel, 1/16” outer diameter, 2 ml (GE p/n 2391650) or 5 ml (GE p/n 2373365)). The loop was sealed by switching V31 and V30. A detailed schematic for the “in-loop” ^11^C-Carbonylation process described above is shown in Figures 2, 3.

### Radiochemistry

*Production of [*^11^*C]CO*. [^11^C]CO_2_ was produced with a GEMS PETTrace cyclotron. The ^14^N(p,α)^11^C nuclear reaction was performed by proton bombardment of a pressurized gas target containing high-purity nitrogen and 0.5% oxygen to generate [^11^C]CO_2_, which was delivered to a GEMS Process Cabinet *via* stainless steel lines (refer to Supplementary Material for more details) (44). [^11^C]CO_2_ was trapped on a molecular sieve column (Grace (p/n 5622) or Ohio Valley Specific Company (p/n 5326), 4Å, 60/80 mesh, 0.4–0.5 g) at room temperature. The accumulated [^11^C]CO_2_ was then released into an online reduction column by heating the molecular sieve trap to 360°C. Two reduction methods were tested to produce [^11^C]CO: (1) [^11^C]CO_2_ was passed through a heated charcoal column (GE coconut charcoal, 20/40 mesh, 2 g, ~6–7 cm in the middle of the 20 cm quartz column) at 950°C, in a stream of helium at flow rates of 40, 250, and 600 ml/min; or (2) [^11^C]CO_2_ was passed through a heated molybdenum column (Alfa Aesar (p/n 3089) or Goodfellow (p/n 802-109-88), 100 mesh powder, 10 g, ~6–7 cm in the middle of the 20 cm quartz column) at 850°C, in a stream of helium at flow rates of 40, 50, and 60 ml/min. The gas was purified through an Ascarite column and delivered out of the GEMS Process Cabinet to a sealed hot cell. The produced [^11^C]CO was trapped on a silica trap (100 mg silica gel in a 1/8 inch tube) immersed in liquid nitrogen and then, after delivery, the [^11^C]CO was released and pushed by helium at 5 ml/min into the HPLC loop for reaction. For comparison of [^11^C]CO yields, the produced [^11^C]CO was passed through a CARULITE 300 column at 650°C to convert it back to [^11^C]CO_2_, which was collected on an Ascarite column. The radioactivity accumulated on the Ascarite column was measured in a dose calibrator, and the yield was calculated based on the initial yields of [^11^C]CO_2_ from the PETtrace that we have previously determined by trapping on the Ascarite columns and measuring in a dose calibrator.

*Using the Loop:* The precursor mixture is loaded onto the loop in the “Load” position by disconnecting the line from the [^11^C]CO trap and slowly injecting the solution from a syringe directly through the valve port. The line is reconnected and the [^11^C]CO enters from the trap while the valve remains in the “Load” position. Following labeling, the six-way valve is then switched to the “Inject” position so the reaction mixture is washed from the loop onto the column by HPLC mobile phase.

*Transfer of [*^11^*C]CO to the HPLC loop*. [^11^C]CO was released from the silica trap by removing it from the liquid N2 trap manually or using a BIMBA pneumatic actuator (Model # FO-094-3CFT) (Figure 3 and Supplementary Figures S11, S12), and pushed by helium at 5 ml/min into the HPLC loop for reaction. Initially, we used two HPLC radioactivity detectors to time the radioactivity transfer time from the silica trap to the HPLC loop. This was established as ~60 s, and this time was programmed into the synthesis module time list for the syntheses described below.

*In-loop radiosynthesis of [*^11^*C]N-benzyl benzamide*. Bis(dibenzylideneacetone)palladium(0) (2 mg, 3.5 μmol) and NiXantphos (2 mg, 3.5 μmol) were dissolved into 200 μl THF and 1.4 μl of iodobenzene was added. The mixture was kept at room temperature for 20 min. Five minutes before the end of the beam, 10 μl of benzylamine was added to the resultant solution. The mixture was vortexed and then loaded onto the HPLC loop. The [^11^C]CO was released from the silica trap into the HPLC loop at a helium flow of 5 ml/min as described above. The loop was sealed for 5 min at room temperature upon completion of this transfer (60 s). Following the labeling reaction, the resulting mixture was injected onto a semi-preparative HPLC column (Prodigy ODS-Prep, 250 × 10 mm) and eluted with 30 mM NH_4_OAc in 50% MeCN/H_2_O at 4 ml/min. The product peak of around 9 min was collected and transferred to a sterile product vial for analysis. HPLC analysis of [^11^C]*N*-benzyl benzamide was performed using an instrument equipped with a radioactivity detector (column: Luna C18 column (150 × 4.6 mm); mobile phase: 30 mM NH_4_OAc in 45% MeCN/H_2_O; flow rate: 1.0 ml/min, UV: 254 nm). The isolated [^11^C]*N*-benzyl benzamide was co-eluted with non-radioactive reference standard and the product was obtained in good radiochemical yields (Mo: 14.1 ± 5.0 GBq, 11.5 ± 4.1%; charcoal: 7.0 ± 3.0 GBq, 5.8 ± 2.4%) and radiochemical purity (Mo: 100 ± 1%; charcoal: 97 ± 2%). The molar activity of [^11^C]*N*-benzyl benzamide was higher when Mo was used to generate [^11^C]CO than activated charcoal (Mo: 306.4 ± 41.5 GBq/μmol; charcoal: 2.1 ± 1.4 GBq/μmol).

*Radiosynthesis of [*^11^*C]Ibrutinib*. Bis(dibenzylideneacetone)palladium(0) (2 mg, 3.5 μmol) and NiXantphos (2 mg, 3.5 μmol) were dissolved into 200 μl THF and 4 μl of vinyl iodide was added. The mixture was kept at room temperature for 20 min. Five minutes before the end of the beam, the solution was transferred to the vial containing 3-(4-phenoxyphenyl)-1-(piperidin-3-yl)-2,3-dihydro-1H-pyrazolo[3,4-d]pyrimidin-4-amine **(2)** (2 mg, μmol). The mixture was loaded onto the HPLC loop. The [^11^C]CO was released from the silica trap into the HPLC loop at a helium flow of 5 ml/min. After transfer for 60 s, the loop was then sealed for 5 min at room temperature. The resulting mixture was purified by semi-preparative HPLC (column: Luna C18 (250 × 10 mm), mobile phase: 0.1% TFA in 50% MeCN/H_2_O; flow rate: 4 ml/min; UV: 254 nm). The product peak of around 9 min was collected into 50 ml water. The solution was loaded through a C18 Sep-Pak cartridge and the cartridge was washed with 10 ml water (USP). The product was eluted with 0.5 ml ethanol (USP) followed by 9.5 ml saline (USP) for injection. The final dose was filtered through a sterile filter (0.22 μm, GV, 13 mm) into a sterile vial. Using this method, 4.7 ± 2.1 GBq (173.9± 77.7 mCi) of [^11^C]ibrutinib was isolated (*n* = 3). Quality control of [^11^C]ibrutinib was conducted according to the guidelines outlined in Chapter <823> of the U.S. Pharmacopeia and previously reported standard procedures (45). HPLC analysis of [^11^C]ibrutinib was performed using an instrument equipped with a radioactivity detector (column: Luna C18 column (150 × 4.6 mm); mobile phase: 30 mM NH_4_OAc in 45% MeCN/H_2_O; flow rate: 1.0 ml/min; UV: 254 nm). The isolated [^11^C]ibrutinib was co-eluted with the non-radioactive reference standard. The sample was >98% radiochemically pure, >90% chemically pure (HPLC, UV: 254 nm), with a molar activity of 281 ± 99 GBq/μmol (7.6 ± 2.7 Ci/μmol), *n* = 3. The overall synthesis time from the end of cyclotron bombardment was 35–40 min.

*Radiosynthesis of [*^11^*C]Tolebrutinib*. Bis(dibenzylideneacetone)palladium(0) (2.7 mg, 4.70 μmol) was dissolved in 300 μl of THF and this solution was added to a sample of NiXantphos 97% (2.7 mg, 4.90 μmol) and the sample was vortexed to dissolve all of the solids for 1 min at ambient temperature. To this solution was added vinyl iodide, 85% (6.24 mg, 3 μl, 0.041 mmol), and the resulting solution was vortexed for an additional minute to ensure proper mixing of the precursor materials. To this solution was added to (R)-4-amino-3-(4-phenoxyphenyl)-1-(piperidin-3-yl)-1,3-dihydro-2H-imidazo[4,5-c]pyridin-2-one **(3)** (1.27 mg, 4.1 μmol) and the crude mixture was vortexed for 30 s. This solution was added to a 5 ml stainless steel HPLC loop before the [^11^C]CO was delivered to the hot cell. [^11^C]CO was transferred from the silica trap using helium gas at 5 ml/min and the transfer time was 60 s. The reaction then occurred within the sealed 5 ml stainless steel HPLC loop at ambient temperature for 5 min. This crude reaction mixture was purified by semi-preparative HPLC (column: Luna C18(2), 5 μ (250 x 9.6 mm); mobile phase: 41% MeCN in 200 mM ammonium formate; flow rate: 5 ml/min; UV: 254 nm). The (R)-[^11^C]-1-(1-acryloylpiperidin-3-yl)-4-amino-3-(4-phenoxyphenyl)-1,3-dihydro-2H-imidazo[4,5-c]pyridin-2-one ([^11^C]tolebrutinib) was isolated between the 13.5 and 15 min mark of the chromatogram and this sample was collected into a dilution flask that contained 55 ml of a 2 mg/ml sodium ascorbate aqueous solution. This solution was transferred to an HLB light (30 mg) SPE cartridge. This cartridge was pre-activated with 5 ml of ethanol followed by 10 ml of sterile water before the synthesis. After transfer, the cartridge was eluted with 0.7 ml of ethanol into the sterile product vial that contained 4 ml of sterile saline for injection. Using this method, 7.1 ± 2.1 GBq (262.7 ± 77.7 mCi) of [^11^C]tolebrutinib was isolated (*n* = 6), and the product was analyzed *via* reverse phase HPLC using the following methods: Method A: column: Zorbax SB C18 3-μm (250 x 4.6 mm); mobile phase A: 0.1% aqueous Trifluoroacetic acid (TFA), mobile phase B: 0.1% TFA in acetonitrile. Gradient method consisting of a solution starting at 5% B and increased to 85% B over a 15-min linear gradient; flow rate: 1.0 ml/min; UV: 254 nm; Method B: Isocratic and molar activity: column: Zorbax SB C18 3 μ (250 x 4.6 mm); mobile phase: Isocratic: 54% acetonitrile in aqueous 0.1% TFA; flow rate: 1.0 ml/min; UV: 254 nm. Method A was used to confirm chemical identity using a co-injection of non-radioactive standard. Radiochemical purity and molar activity were determined by Method B. [^11^C]Tolebrutinib was confirmed by co-injection with a verified non-radioactive reference standard. *A*_m_ was determined using a 6-point standard curve (analytical HPLC peak area (Y) vs. standard concentration (X: in nmol)) by comparison with a reference standard of known concentration (1.25 mg in 50 ml). The isolated [^11^C] tolebrutinib was co-eluted with a non-radioactive reference standard. The sample was 99.9% radiochemically pure, 97% chemically pure (HPLC, UV: 254 nm), with a molar activity of 500 ± 33 GBq/μmol (7.1 ± 2.1 Ci/μmol), *n* = 6. The overall synthesis time from the end of cyclotron bombardment was 33–35 min.

*Radiosynthesis of [*^11^*C]Evobrutinib*. [^11^C]Evobrutinib was synthesized similarly to the Tolebrutinib example above with the following exceptions. First, the precursor 5-(4-phenoxyphenyl)-N4-(piperidin-4-ylmethyl)pyrimidine-4,6-diamine (4) (1 mg, 2.7 μmol) was used and the crude reaction mixture after the carbonylation reaction was purified by semi-preparative HPLC (column: Luna C18(2), 5 μ (250 x 9.6 mm); mobile phase: 44% MeCN in 200 mM ammonium formate; flow rate: 5 ml/min; UV: 254 nm). The [^11^C]1-(4-(((6-amino-5-(4-phenoxyphenyl)pyrimidin-4-yl)amino)methyl)piperidin-1-yl)prop-2-en-1-one ([^11^C]evobrutinib) was isolated between the 15.5 and 18 min mark of the chromatogram and this sample was collected into a dilution flask that contained 50 ml of a 2 mg/ml sodium ascorbate aqueous solution. This solution was transferred to an HLB light (30 mg) SPE cartridge. After transfer, the cartridge was eluted with 1 ml of ethanol into the sterile product vial that contained 4 ml of sterile saline. Using this method, 2.2 ± 0.6 GBq (81.4 ± 22.2 mCi) [^11^C]evobrutinib was isolated (*n* = 3), and the product was analyzed *via* reverse phase HPLC using the following methods. Method A described above and Method B (Isocratic and molar activity): column: Luna C18(2) 3-μm (250x4.6 mm); mobile phase Isocratic: 36% acetonitrile in aqueous 0.1% TFA; flow rate: 1.3 ml/min; UV: 254 nm. Method A was used to confirm chemical identity using a co-injection of non-radioactive standard. Radiochemical purity and molar activity were determined by Method B. [^11^C]Evobrutinib was confirmed by co-injection with a verified non-radioactive reference standard. *A*_m_ was determined using a 4-point standard curve (analytical HPLC peak area) (Y) vs. standard concentration (X: in nmol) by comparison with an evobrutinib reference standard of known concentration (2.3 mg in 1 ml). The isolated [^11^C] evobrutinib was co-eluted with a non-radioactive reference standard. The sample was >99% radiochemically pure, >95% chemically pure (HPLC, UV: 254 nm), with a molar activity of 496.5 ± 74 GBq/μmol (13.4 Ci/μmol) The overall synthesis time from the end of cyclotron bombardment was 37–46 min.

## Results and Discussion

In this study, our team employed methods to incorporate a carbon-11 radionuclide into the acrylamide moiety of several clinical BTK inhibitors with high radiochemical yields and high molar activities. Ibrutinib, tolebrutinib, and evobrutinib all lend themselves for [^11^C]CO carbonylation utilizing a new Pd/NiXantphos-mediated methodology. To accomplish this, our team focused first on the production of [^11^C]CO and modifications to commercially available remote-controlled synthesis units, and optimization of labeling using a model substrate.

Two methods of producing [^11^C]CO were investigated. Both an activated charcoal and molybdenum column were installed in our [^11^C]CO synthesizer prototype (GEMS Process Cabinet). [^11^C]CO_2_ was either passed through a heated activated charcoal column (950°C) or through a heated molybdenum column (850°C) using a stream of helium to produce [^11^C]CO. These two methods produced comparable isolated yields of [^11^C]CO: the radiochemical yield of [^11^C]CO was 78.4 ± 4.7% (*n* = 6) at the end of synthesis for the charcoal method, while the molybdenum method (using 100 mesh) gave radiochemical yields of 69.1 ± 7.6% (*n* = 6). Substantially lower yields were obtained using 20 mesh Mo (<10%). Three helium flow rates of 40, 100, and 600 ml/min were tested. The fastest flow rate gave a slightly higher radiochemical yield due to shorter delivery time (~2 min) from process cabinet to hot cell, while the slowest flow took about 6 min, but no significant effects from varying flow rates were observed.

With satisfactory methods for producing [^11^C]CO established, we next investigated the synthesis of [^11^C]*N*-benzyl benzamide as a model reaction to evaluate ^11^C-carbonylation using [^11^C]CO produced *via* both methods (Scheme 2A). To facilitate this comparison, both a GE TracerLab FX_M_ and FX_C−Pro_ synthesis modules were modified for in-loop ^11^C-carbonylation as shown in Figure 2. Notably, while Ferrat et al. showed that higher yields could be obtained at elevated temperatures (39), all ^11^C-carbonylation reactions in this study were conducted at room temperature. This was for operational simplicity as it negated the need to install a heater for the synthesis module HPLC loop. Radiochemical yields were suitable for clinical use (*vide infra*), but there is scope for additional improvements if a heating device that offers adequate temperature control can be installed. A homemade silica column was used to [^11^C]CO trap generated from either charcoal or molybdenum methods. This silica trap was easily made from a 1/8th inch (either polytetrafluoroethylene or stainless steel) tubing that contained ~100 mg of silica (55–105 μm) and gave excellent trapping efficiency (>90%) when immersed in liquid nitrogen for 10 min before the end of the beam and released >95% of the radioactivity when moved out of liquid nitrogen without any extra heating. As shown in Figure 3, [^11^C]CO was released from the silica trap to the HPLC loop in ~60–90 s and delivered to position 3 of an HPLC switching valve in the load position A. This allowed the [^11^C]CO to flow directly into a 2 or 5 ml stainless steel HPLC loop that contained the benzylamine precursor, iodobenzene, Pd catalyst and XantPhos ligand for the “in-loop” ^11^C-carbonylation of [^11^C]*N*-benzyl benzamide (1) (Scheme 2A).

Based on a previous report (39), 2.5 mg Pd_2_(π-cinnamyl)Cl_2_ and 5.0 mg XantPhos were first tested. A 700 μl of THF was needed to dissolve the catalysts, ligand, and iodobenzene. The solvent was fully evaporated, and the residue was redissolved into 150 μl of dioxane. After adding benzylamine, precipitation was observed. To prevent clogging of the HPLC loop, injector, and lines, filtration was required before loading the reaction mixture onto the HPLC loop. Although the radiochemical conversion from this procedure was acceptable (61 ± 12%, *n* = 5), the results were not reliable. The volume and concentration of the reaction mixture were not consistent for each run due to precipitation and filtration. In addition, when using an organohalide with low a boiling point, like vinyl iodide, reagent loss would be expected during the evaporation step. Moreover, from our experiences with loop chemistry using 2 ml stainless steel HPLC loops on TracerLab FX_M_, a volume between 100 and 200 μl gives the best results (46). Volumes larger than 200 μl cause lower yields due to the loss of reaction mixture from the other end of the loop. However, even reducing the ligand concentration from 5 to 2 mg, 500 μl of THF was needed to fully dissolve all the reagents. Thus, we considered more soluble catalysts and ligands to further optimize the chemistry.

Vasdev's group recently reported high yielding ^11^C-carbonylation procedures using bis (dibenzylideneacetone) palladium(0) (Pd(dba)_2_) and NiXantphos (28). Both Pd(dba)_2_ and NiXantphos are more soluble in THF compared to Pd_2_(π-cinnamyl)Cl_2_ and XantPhos. Thus, Pd(dba)_2_ (2 mg, 3.5 μmol) and NiXantphos (2 mg, 3.5 μmol) were dissolved in THF (200 μl), and iodobenzene (1.4 μl) was added. The mixture was kept at ambient temperature for 20 min and 5 min before the end of the beam, benzylamine (10 μl) was added. The mixture was loaded onto the HPLC loop and [^11^C]CO was released from the silica trap into the HPLC loop. The loop was then sealed for 5 min at room temperature after which time the crude reaction mixture was injected and purified by semi-preparative HPLC to give [^11^C]*N*-benzyl benzamide (**1**) in similar isolated radiochemical yields (Mo: 14.1 ± 5.0 GBq, 11.5 ± 4.1%; charcoal: 7.0 ± 3.0 GBq, 5.8 ± 2.4%) and radiochemical purity (Mo: 100 ± 1%; charcoal: 97 ± 2%), although in each instance the Mo method worked slightly better. The semi-preparative HPLC trace showed a large radiochemical impurity peak formed when using the charcoal method likely leading to the slightly lower radiochemical purity (Supplementary Figure S1A), while a much cleaner trace was observed using the molybdenum method (Supplementary Figure S1B). The molar activity of [^11^C]*N*-benzyl benzamide was substantially higher when Mo was used to generate [^11^C]CO than activated charcoal (Mo: 306.4 ± 41.5 GBq/μmol; charcoal: 2.1 ± 1.4 GBq/μmol). This is not unexpected as various impurities are occurring on the surface of activated charcoal (47). The manufacturing process of activated charcoal produces large amounts of oxygen-containing groups on the outer surface of charcoal (e.g. carbonyl, carboxylic, lactone, and phenol groups) that can lead to isotopic dilution (48, 49). It should be noted there was a significant cold mass peak within the UV HPLC trace using charcoal (Supplementary Figure S1A) that was consistent with extremely low molar activity. This suggested that the cold mass was not only from isotope dilution from charcoal but possibly also from the decomposition of carbonyl groups of the impurities, generating non-radioactive carbon monoxide and/or carbon dioxide (50). The CO from residual acids, esters, ketones, or aldehydes might be reduced by heating the column under inert gas for a longer time, but we believe the carbon-12 from the charcoal will still lead to isotope dilution. This in conjunction with the higher radiochemical yields, radiochemical purities, and molar activities obtained using [^11^C]CO derived from a molybdenum reduction method, clearly established this as the optimal method to use to investigate the radiosynthesis of ^11^C-labeled BTK inhibitors.

Using the optimized conditions for “in-loop” ^11^C-carbonylation, [^11^C]ibrutinib, [^11^C]tolebrutinib, and [^11^C]evobrutinib were produced under conditions amenable for clinical use. In each instance Pd(dba)_2_ (2.0-2.7 mg) and NiXantphos (2.0–2.7 mg) were dissolved in THF (200–300 μl), and vinyl iodide (3–4 μl) was added. The mixture was vortexed and kept at room temperature. Five minutes before the end of the beam, the appropriate amino precursor (1–2 mg) was added (Scheme 2). The mixture was vortexed again and added to the appropriate HPLC loop. [^11^C]CO was released from the silica trap into the HPLC loop. The loop was then sealed for 5 min at ambient temperature for reaction to occur, after which time the crude reaction mixture was injected and purified by semi-preparative HPLC (refer to Supplementary Material for representative HPLC traces) to yield the ^11^C-labeled BTK inhibitors. This single-step reaction and short synthesis procedure (~40 min) produced the radioligands in good radiochemical yields and high molar activity (Table 1). The radiopharmaceuticals were isolated in >95% radiochemical purity, and molar activities ranged from 281–500 GBq/μmol (7.5–13.0 Ci/μmol).

**Table 1:** Radiosynthesis of ^11^C-labeled BTK inhibitors.

**Compound**	**Isolated yield** **(GBq)**	**A_**m**_** **(GBq/mol)**	**RCY** **(non-decay corrected)**	***N***
[^11^C]Ibrutinib	4.7 ± 2.1	281 ± 99	3.3 ± 1.5%	3
[^11^C]Tolebrutinib	7.1 ± 2.1	500 ± 33	12.8 ± 2.1%	6
[^11^C]Evobrutinib	2.2 ± 0.6	497 ± 74	3.9 ± 0.6%	3

Finally, encouraged by these results, we validated the synthesis of [^11^C]ibrutinib to confirm suitability for clinical use. Three process verifications runs were completed and produced [^11^C]ibrutinib in 3.3 ± 1.5% radiochemical yield (based on [^11^C]CO_2_, non-decay corrected), >98% radiochemical purity and molar activity of 7.6 ± 2.7 Ci/μmol (281 ± 99 GBq/μmol), *n* = 3 (Table 2). Quality control testing of [^11^C]ibrutinib was conducted according to the guidelines outlined in Chapter <823> of the U.S. Pharmacopeia, and previously reported QC procedures (refer to Supplementary Material for representative HPLC traces) (29, 45). As shown in Table 2, each batch of [^11^C]ibrutinib met all acceptance criteria confirming suitability for future clinical studies.

**Table 2:** Quality control data of [^11^C]Ibrutinib validation radiosyntheses.

**QC test**	**Acceptance criteria**	**Batch 1 result**	**Batch 2 result**	**Batch 3 result**	**Pass/fail**
Radiochemical purity	NLT 90%	98%	99%	99%	Pass
Radioactive concentration	NLT 40 mCi/10 mL @EOS	172 mCi/10 mL	51 mCi/10 mL	159 mCi/10 mL	Pass
Active ingredient concentration	Report results (μg/mL)	1.1 μg/mL	0.21 μg/mL	1.2 μg/mL	Pass
Molar activity	≥1,000,mCi/μmol	6,429 mCi/μmol	10,729 mCi/μmol	5,688 mCi/μmol	Pass
pH	4.5–7.5	5.5	5.5	5.5	Pass
Visual inspection	Clear, colourless, no ppt	Clear, colourless, no ppt	Clear, colourless, no ppt	Clear, colourless, no ppt	Pass
Radiochemical identity (HPLC)	RRT: 0.9–1.1	1.04	1.04	1.03	Pass
Radionuclide identity	18.4–22.4 min	20.15 min	19.89 min	20.1 min	Pass
Filter membrane integrity	≥45psi	48 psi	49 psi	49 psi	Pass
Bacterial endotoxin	≤ 17.5 EU/mL	<2.00 EU/mL	<2.00 EU/mL	<2.00 EU/mL	Pass
Residual solvent analysis	MeTHF/THF ≤ 720 ppm MeCN ≤ 410 ppm	THF <2 ppm MeCN 29 ppm	THF <2 ppm MeCN <2 ppm	THF <2 ppm MeCN 42 ppm	Pass
**Post-release QC test**	**Release criteria**	**Batch 1 result**	**Batch 2 result**	**Batch 3 result**	**Pass/fail**
Sterility	Sterile	Sterile	Sterile	Sterile	Pass

## Conclusions

A simple and high yielding ^11^C-carbonyl labeling process is described. The ^11^C-carbonylation is carried out “in the loop” at room temperature without the need for specialized high-pressure or high-temperature equipment. The automated method uses standard commercially available radiosynthesis modules that are routinely used for ^11^C-methylation. The simple modifications described herein enable the cGMP production of radiopharmaceuticals such as radiolabeled BTK inhibitors. As a proof-of-concept, [^11^C]ibrutinib has been synthesized in good radiochemical yield, with high radiochemical purity and molar activity. Quality control testing confirmed the suitability of the radiotracer for clinical use. We anticipate that this method will be applicable to many additional PET tracers for preclinical and clinical applications in the future. This, in conjunction with the operational simplicity of the method and compatibility with routinely used and commercially available equipment, has the potential to enable routine use of ^11^C-carbonylation for the synthesis of PET radiotracers by any laboratory with the means of producing [^11^C]CO.

## Data Availability Statement

The original contributions presented in the study are included in the article/Supplementary Material, further inquiries can be directed to the corresponding author/s.

## Author Contributions

XS, DD, and PS designed the research and wrote the manuscript. DD, DB, TW, TK, SP, KM, BH, and XS performed research. TK, BH, XS, TW, DD, and PS analyzed the data. All the authors reviewed and approved the manuscript.

## Conflict of Interest

DD, TW and DB are, and TT was employed by the Bristol Myers Squibb Research and Development. The remaining authors declare that the research was conducted in the absence of any commercial or financial relationships that could be construed as a potential conflict of interest.

## Publisher's Note

All claims expressed in this article are solely those of the authors and do not necessarily represent those of their affiliated organizations, or those of the publisher, the editors and the reviewers. Any product that may be evaluated in this article, or claim that may be made by its manufacturer, is not guaranteed or endorsed by the publisher.
