# Supplementary material for: Synthesis of Radiopharmaceuticals via “In-Loop” 11C-Carbonylation as Exemplified by the Radiolabeling of Inhibitors of Bruton's Tyrosine Kinase
Source: Front Nucl Med. 2022 Jan 20;1:820235. doi: 10.3389/fnume.2021.820235 (PMC11440948; doi:10.3389/fnume.2021.820235)
Supplement: Supplementary file 1 [file Data_Sheet_1.pdf]

Synthesis of radiopharmaceuticals via “in-loop” ^{11}C-carbonylation as exemplified by the radiolabeling of inhibitors of Bruton’s Tyrosine Kinase

Table of Contents

1. HPLC Traces for the Radiosynthesis of [^{11}C]N-benzyl benzamide S2
2. HPLC Traces for the Radiosynthesis of [^{11}C]Ibrutinib S5
3. HPLC Traces for the Radiosynthesis of [^{11}C]Tolebrutinib S7
4. HPLC Traces for the Radiosynthesis of [^{11}C]Evobrutinib S9
5. Photographs of experimental set-up S11

1. HPLC Traces for the Radiosynthesis of [^{11}C]N-benzyl benzamide

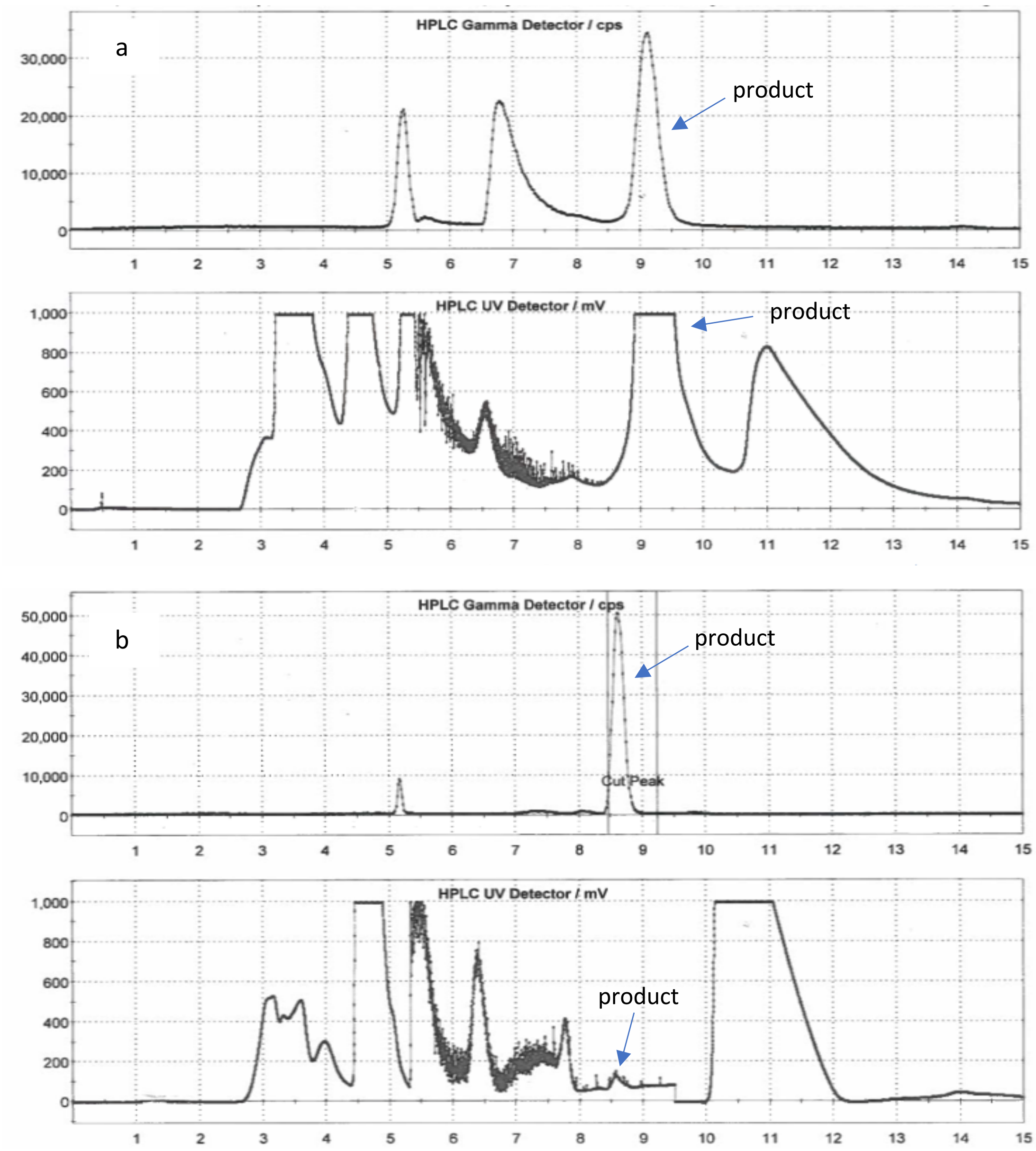

Figure S1. Semi-preparative HPLC traces for [^{11}C]N-benzyl benzamide. (a) Charcoal method; (b) Molybdenum method.

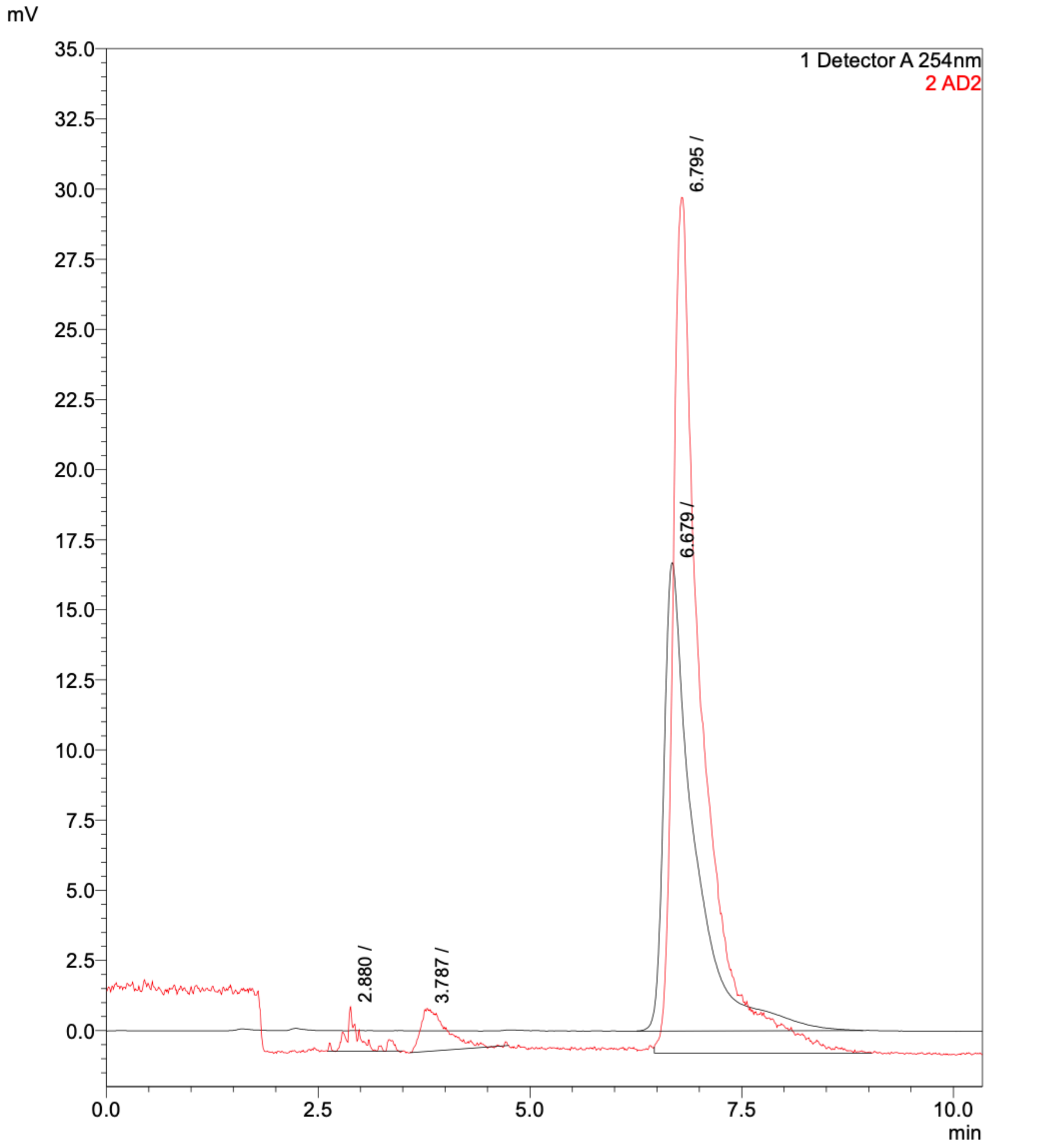

Figure S2. Analytical radio-HPLC chromatogram (Red) of [^{11}C]N-benzyl benzamide prepared using the charcoal method, co-injected with non-radioactive reference standard (Black)

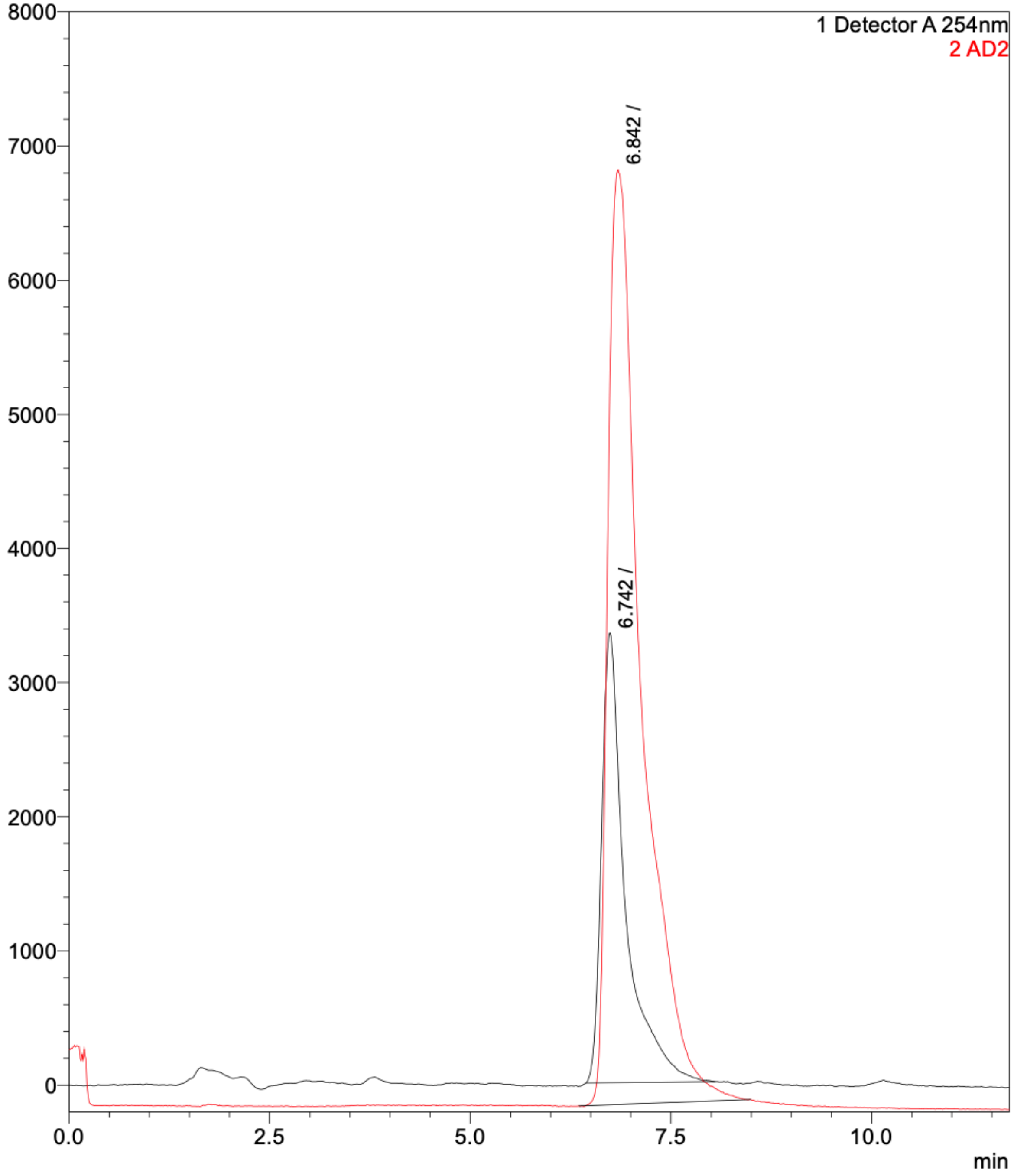

Figure S3. Analytical radio-HPLC chromatogram (Red) of [^{11}C]N-benzyl benzamide prepared using the molybdenum method, co-injected with non-radioactive reference standard (Black)

2. HPLC Traces for the Radiosynthesis of [^{11}C]Ibrutinib

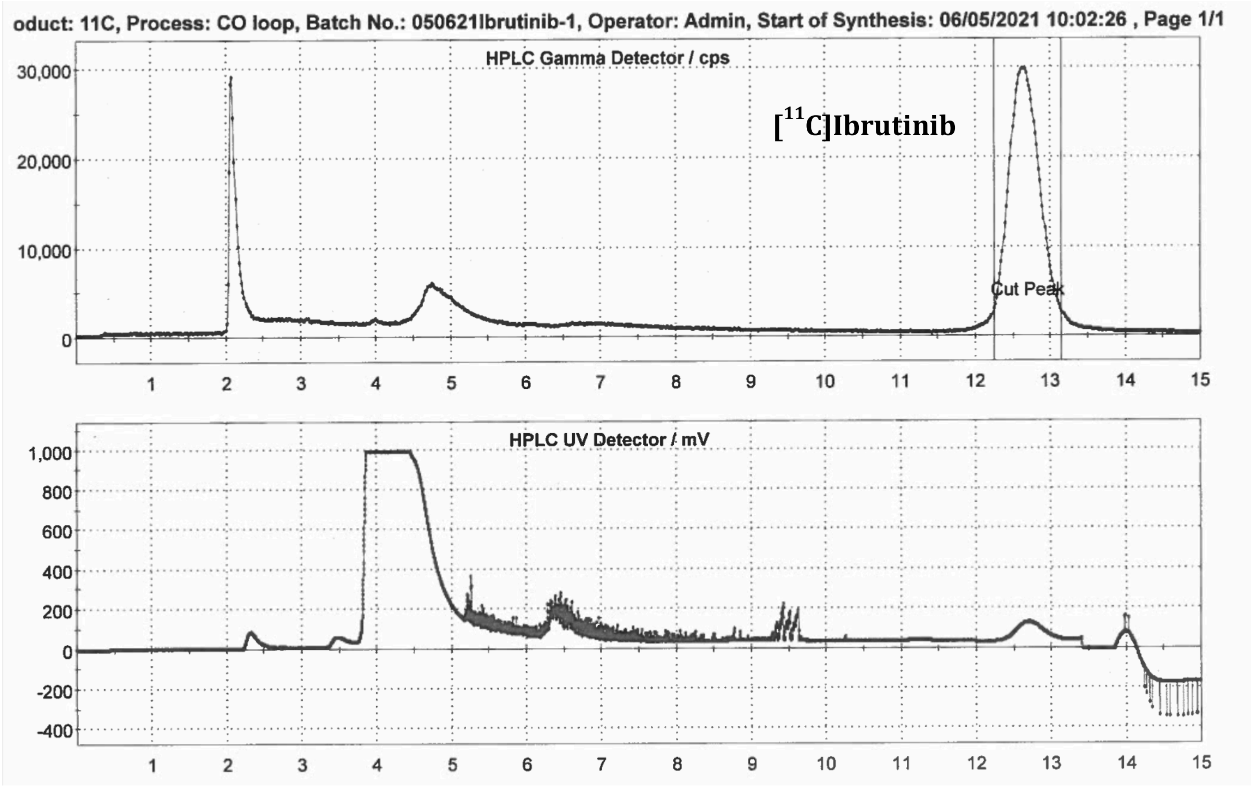

Figure S4: Semi-preparative HPLC Chromatogram of [^{11}C]Ibrutinib

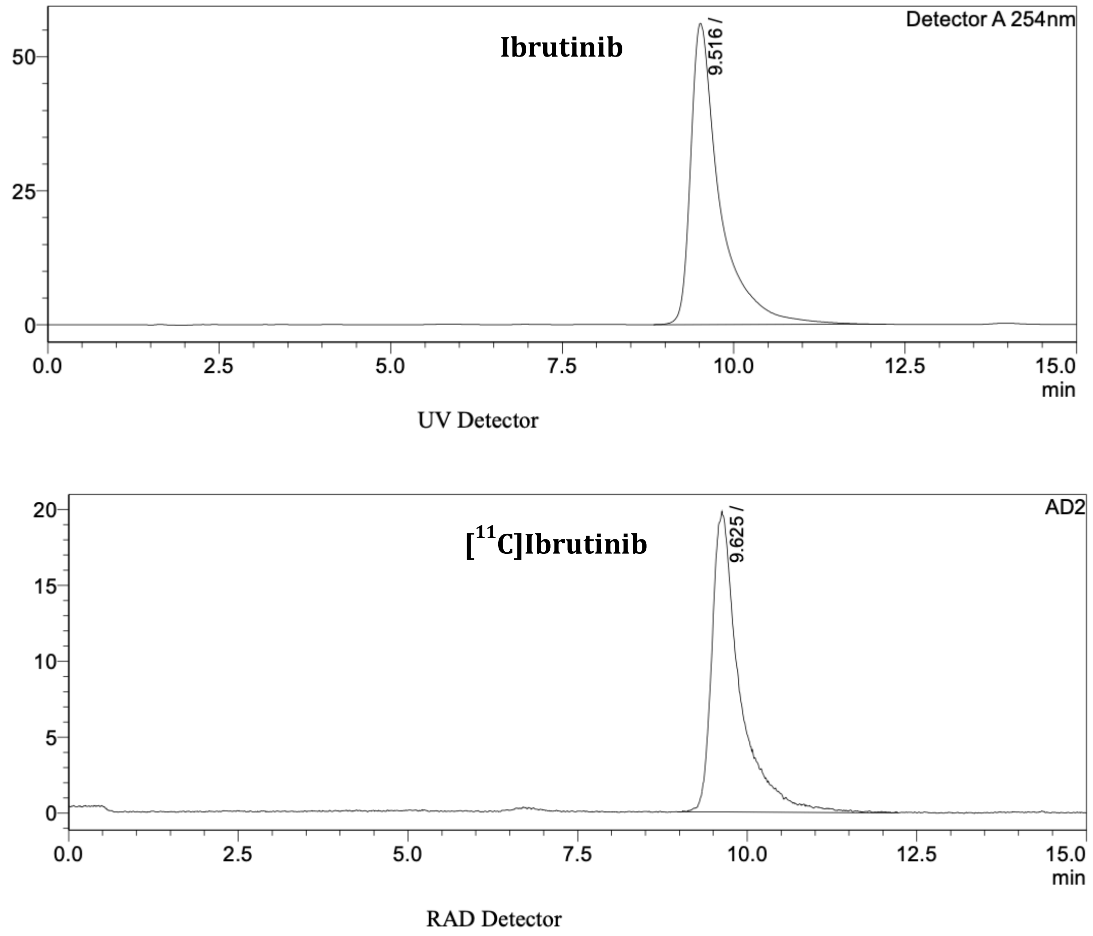

Figure S5: Analytical radio-HPLC chromatogram of [^{11}C]ibrutinib (Bottom) co-injected with non-radioactive reference standard (Top)

3. HPLC Traces for the Radiosynthesis of [^{11}C]Tolebrutinib

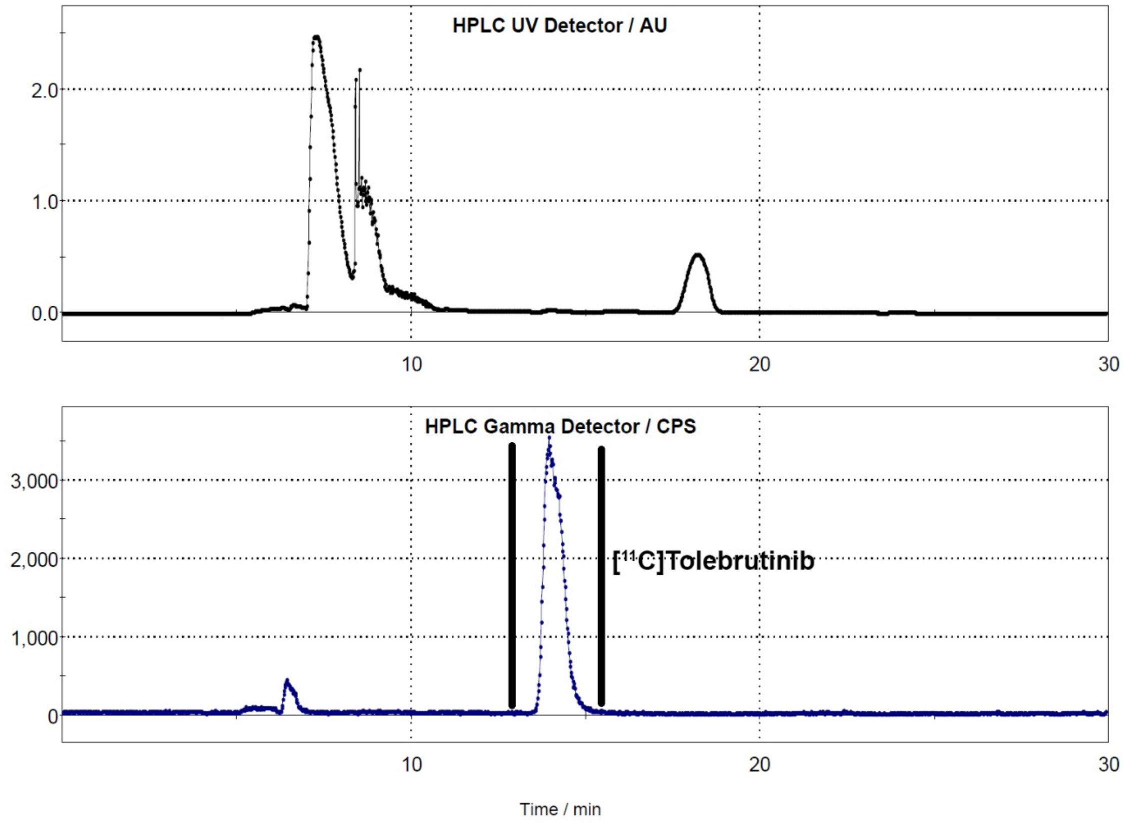

Figure S6: Semi-preparative HPLC Chromatogram of [^{11}C]Tolebrutinib

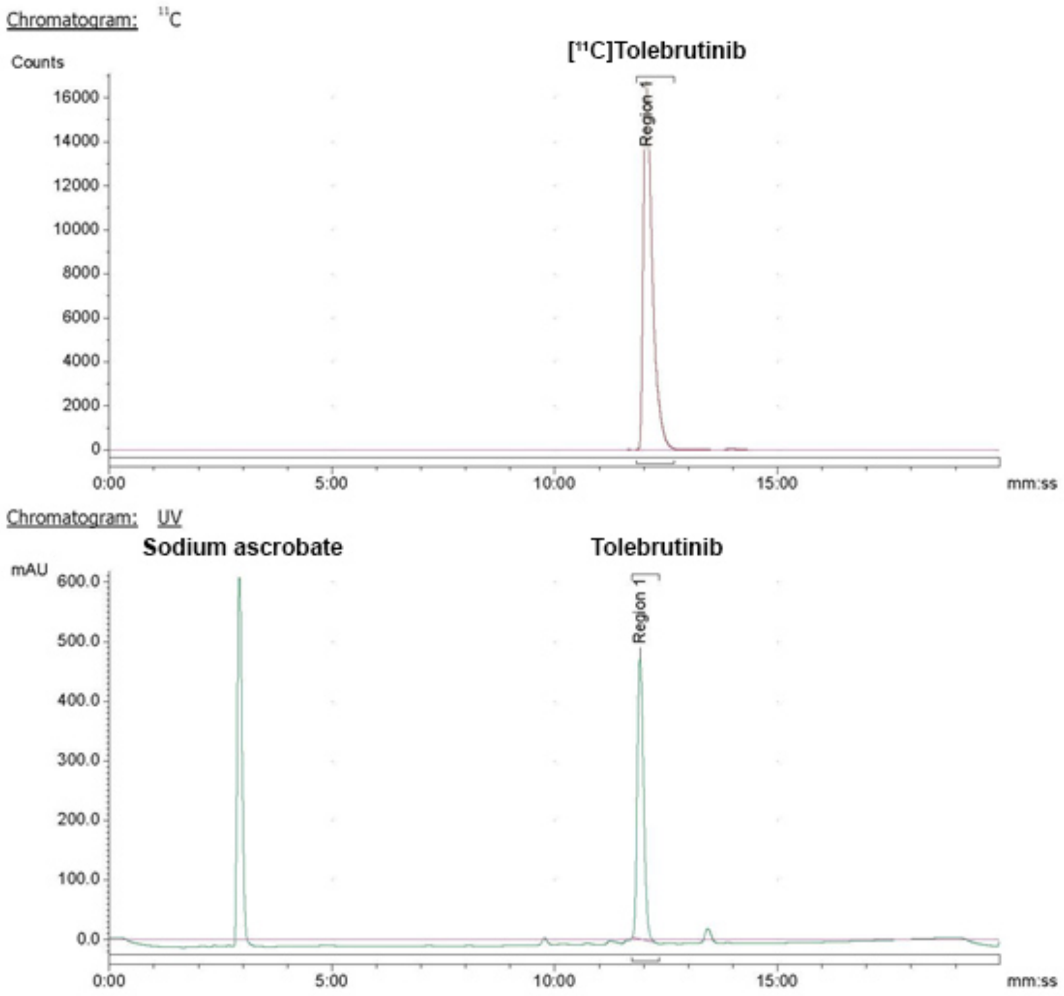

Figure S7: Analytical radio-HPLC chromatogram of [^{11}C]tolebrutinib (Top) co-injected with non-radioactive reference standard (Bottom)

4. HPLC Traces for the Radiosynthesis of [^{11}C]Evobrutinib

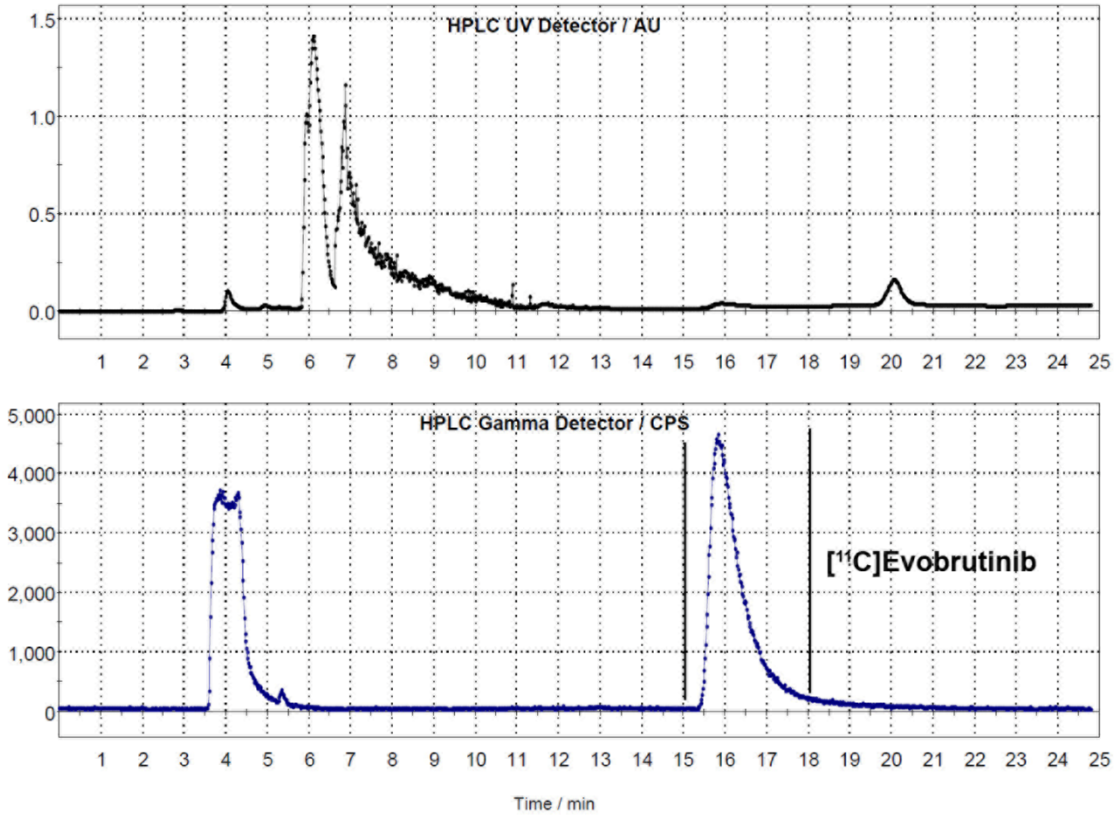

Figure S8: Semi-preparative HPLC Chromatogram of [^{11}C]Evobrutinib

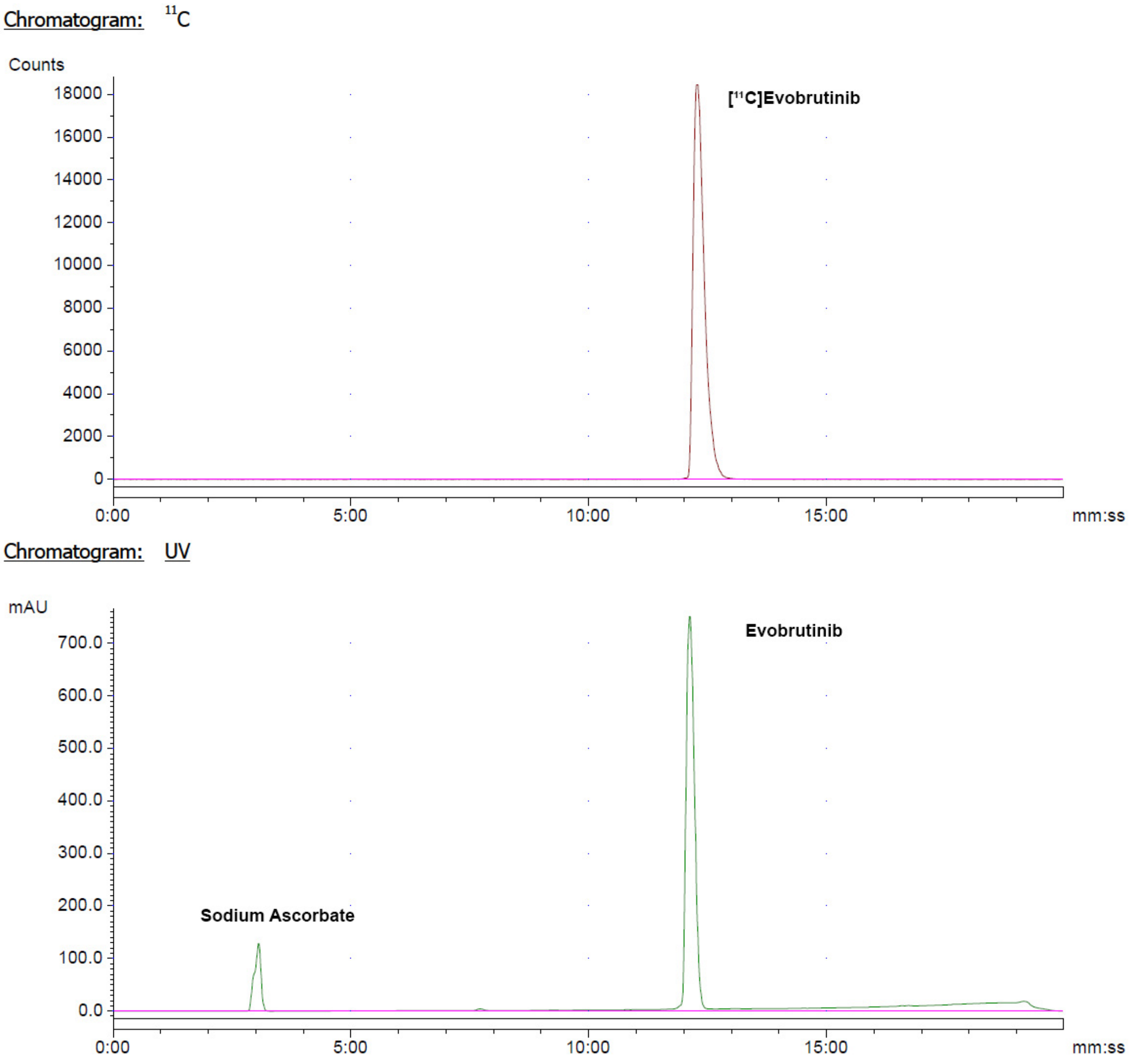

Figure S9: Analytical radio-HPLC chromatogram of [^{11}C]evobrutinib (Top) co-injected with non-radioactive reference standard (Bottom)

5. Photographs of Experimental Set-up

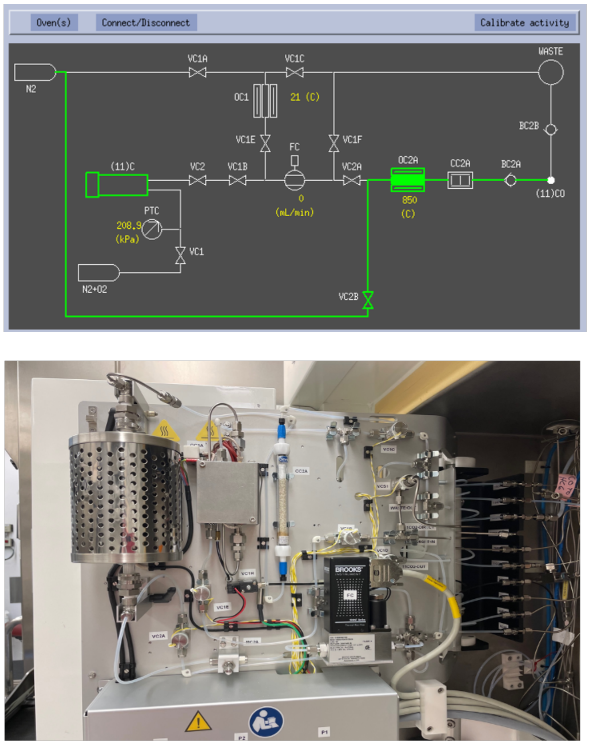

[^{11}C]CO:

Beam duration: 2 minutes

Yield:

348 ± 21 mCi (charcoal, 950°C)

307 ± 34 mCi (molybdenum, 850°C)

Flow rate options:

(a) 40 – 600 mL/min (charcoal)

(b) 40-60 mL/min (molybdenum)

(c) use cold trap 1.5– 40 mL/min

Figure S10: GEMS Procab setup and interface.

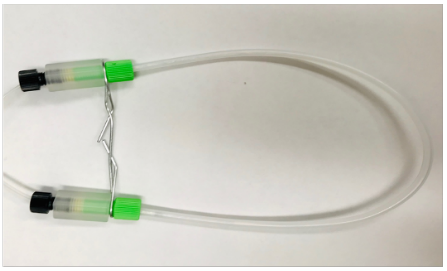

A 12-inch-long tube (1/8”) was filled with about 3 inches of glass wool at each end and 100 mg of silica (from Sep-Pak plus) in the middle (about 5 inches).

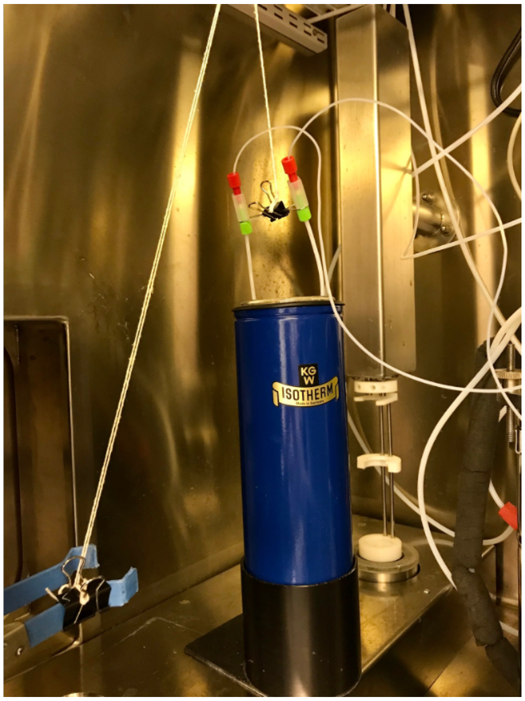

Figure S11: Manual-controlled silica trap for [^{11}C]CO

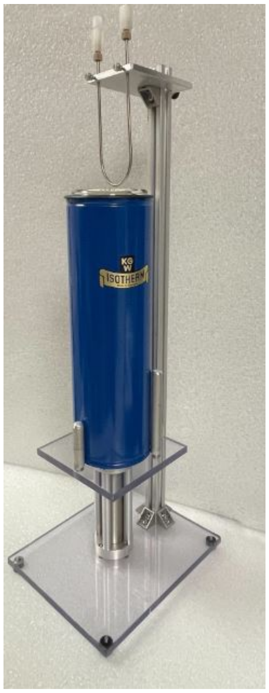

Figure S12: Pneumatic actuator-controlled silica trap for [^{11}C]CO

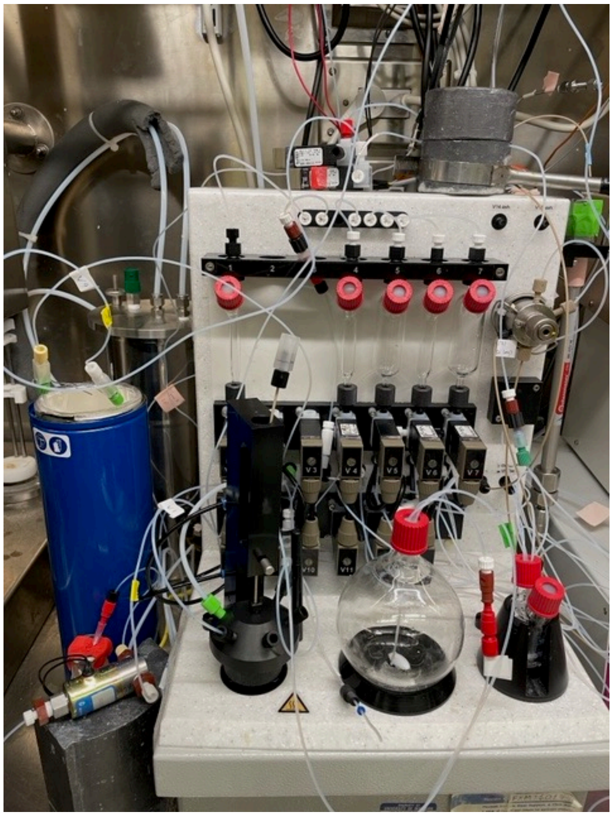

Figure S13: Silica trap for [^{11}C]CO connected to HPLC loop of TRACERLab Synthesis Module
